# The COVID-19 pandemic: when science collided with politics, culture and the human imagination

**DOI:** 10.1098/rsfs.2021.0070

**Published:** 2021-10-12

**Authors:** Roger Highfield

**Affiliations:** Science Museum Group, London, UK

As the SARS-CoV-2 virus continues to disrupt life around the world, the pandemic has provided a mirror with which to review the relationship between science, policymaking and society. It reflects the more successful aspects of the response to COVID-19, such as the remarkable speed of vaccine development, some perplexing features, such as anti-vaccine sentiment, the efficacy, public acceptance and political influence of epidemiology, and more.

There is no better perspective to gaze through this looking glass than from the viewpoint of the Science Museum Group, a cultural institution which acts as a nexus for government, industry, the charitable sector and the public, along with the past, present and future of science, engineering and innovation [1].

Museums can offer unique perspectives on the spread of infectious disease [2]. Their collections and scholarship reveal the lessons of the past, such as the historic debates over the benefits and risks of vaccination [3]. Moreover, through collecting contemporary objects along with exhibitions and events, museums can shed light on how science can shape our future, whether through the development of new therapeutics, monitoring the evolution of a virus, or by modelling. They can also show how we, in turn, can shape science.

The pandemic has also driven the evolution of museums, compelling them along with many other organizations to engage with audiences online and to go beyond traditional ‘material culture’, where stories are told through objects, to find new ways to inform audiences about the threat posed by this invisible enemy and the scientific response.

To reflect the greatest global health crisis in a generation, the five museums that form the Science Museum Group have launched their largest ever collecting project, which has acquired the first doses of COVID vaccine given in the UK, testing kits and the signs used in government briefings among many other things.

The Group has hosted a series of well-attended virtual events, involving leading figures such as Anthony Fauci, chief medical advisor to the President of the United States of America; Sarah Gilbert of the Jenner Institute, University of Oxford; Kate Bingham former chair of the UK Government's Vaccine Taskforce; and Chris Whitty [4], Chief Medical Officer to England. The Group has published more than 120 000 words about COVID-19 in blogs that aimed to share the latest expert knowledge with the public on a range of themes, from the use of organoids [5] and AI [6] to the rollout of vaccines [7].

The Science Museum itself hosted the world's first Global Vaccine Confidence Summit and an NHS vaccination centre [8], where thousands of people were inoculated, including the Health Secretary and the Duke and Duchess of Cambridge. The group is now working on an exhibition about the hunt for an effective COVID-19 vaccine with the National Museums of Scotland, National Council of Science Museums India, and the Guangdong Science Centre and its network in China.

Both this journal and the museums act as a melting pot for ideas. One focus on this issue is the interface between the pandemic, policy and the machinery of government, which has been a preoccupation for millennia: Cicero was prompted to remark, *Salus populi suprema lex esto* (‘The health (welfare, good, salvation, felicity) of the people should be the supreme law’).

In his contribution along these lines to this issue of *Interface Focus*, Chris Whitty, Chief Medical Officer, and Luke Collet-Fenson examine what COVID-19 can tell us about formal and informal science advice in emergencies, and the tensions that emerge, notably between comprehensive advice that has been rigorously tested against speed, along with striking the right balance between taking on board diverse views and groupthink. Adapting the existing structures of scientific advice is more effective than creating new ones in an emergency and, while a final judgement of the UK scientific response will take time, and that judgement is likely to evolve, they argue that everyone should be grateful to the thousands of scientists involved in the pandemic effort.

Philip Ball takes a more critical view, showing how the COVID-19 pandemic has highlighted—as never before—how science works and the ways in which science interacts with policymaking and with society [9]. He notes that how well a country has fared in avoiding illness and fatalities is, roughly speaking, uncorrelated with either its wealth or its scientific strength. There are lessons for scientists and politicians, he concludes, and the latter should acknowledge that scientific advice is likely to be more effective when it is genuinely independent, autonomous and transparent.

One draconian aspect of government responses—stay at home orders—is examined by Toby Phillips, Yuxi Zhang and Anna Petherick [10]. Drawing on data from the Oxford COVID-19 Government Response Tracker, they reveal three broad trends in their use of non-pharmaceutical interventions in the first year that pose questions about the extent to which pandemic management depends on early decisions. They conclude that seeking to make sense of tendencies in non-pharmaceutical intervention adoption, while vaccination programmes spread globally gradually and inconsistently, could assist policymakers in making better decisions to overcome this and future global health crises.

Fiona Watt, who is the Executive Chair of the UK's Medical Research Council, with Patrick Chinnery, Jonathan Pearce, Anna Kinsey, Joanna Jenkinson and Glenn Wells, look back at how UK government support for COVID-19 medical research evolved. This was primed by previous experience with Ebola and Zika, beginning with the early calls for proposals in February 2020 that ‘pump-primed’ funding for vaccines and therapeutics, and culminating in the launch of the government's National Core Studies programme in October.

They discuss how the research community mobilized to submit and review grants more rapidly than ever before, despite laboratory and office closures caused by the pandemic, and highlight the challenges of running clinical trials as the number of hospitalized patients fluctuated with different waves of the disease. The pandemic response has already left an important legacy, which ranges from a UK vaccines manufacturing capacity to a helpful blurring of interests in those in applied and discovery medical research.

Like Watt *et al*. [11] Jim Smith and David Goodhew [12] argue that the urgency and focus imposed by COVID-19 prompted funders to become nimble and also benefited from a ‘seed corn’ of discovery science, from the basis for routine diagnostic tests to the development of vaccines [11]. The speed of dissemination of research has benefitted from the widespread use of pre-prints, such as from *bioRvix* and *medRxiv*, which present an open and rapid way to share pre-peer reviewed studies. But the advice provided to schools on the basis of this research was, however, often published at the last minute, flawed or inconsistent. Their report concludes: ‘Must do better’.

One issue of contention early in the pandemic was the effectiveness of face coverings given the lack of evidence from randomized control trials. Lydia Bourouiba, Katherine Randall, E. T. Ewing, Linsey Marr and Jose Jimenez explore how the pandemic exposed major gaps in our understanding of the transmission of viruses through the air which slowed recognition of airborne transmission of COVID-19 and contributed to muddled public health policies and messaging [13]. They revisit the past to highlight potential future solutions and argue the importance of using a historical perspective to help design more resilient, far-sighted and effective public health policies.

In the light of the debate about the effectiveness of face coverings, Trish Greenhalgh [14] examines how mental models have sometimes facilitated the thinking of scientists and other times been a hinderance. The latter occurred in the case of COVID-19 when undue emphasis was initially placed on randomized control trials, which were inconclusive at the start of the pandemic, when it was also believed that the disease was spread by respiratory droplets.

By June 2020, more than 200 aerosol scientists argued that mechanistic evidence had demonstrated ‘beyond any reasonable doubt’ that the SARS-CoV-2 virus is carried long distances by microdroplets. Models and empirical thinking are complementary but, by not fully appreciating how they work hand in glove, scientists and policymakers initially favoured experimental evidence over theory based on mechanistic insights, rejecting the use of face coverings. In particular, the World Health Organization was sluggish to respond to emerging evidence. As a result, lives were lost.

The successful development of COVID-19 vaccines has outpaced the production of antiviral drugs. There is a bottleneck when screening vast numbers of potential small molecules (ranging from a few hundred million to billions) to shortlist lead compounds for COVID-19 antiviral drug development. To overcome this hurdle, I discuss with Peter Coveney and colleagues [15] from a diverse and international range of institutions how to use a judicious combination of *in silico* theory-led modelling with AI methods that rely on big data [16]. At the interface between AI, in the form of machine learning methods, and physics-based methodology, each compensates for the weaknesses of the other. Together they offer a way to reform the drug discovery process, which is expensive, inefficient and slow, to deliver pandemic drugs at pandemic speed.

Aside from the direct impact of the pandemic, there has been considerable speculation regarding how people coped with the health crisis, and to what extent. Adam Hampshire, Peter Hellyer, William Trender and Samuel Chamberlain describe an unbiased approach that learns from people's collective lived experiences through the application of AI in the form of natural-language processing of free-text reports [17]. Based on an analysis of texts about impact and means of coping from more than 50 000 UK individuals in the first lockdown, they concluded that 45 topics were required to optimally summarize practical coping strategies that they recommended, and that the relevance of the topics could be predicted from population variables such as age. They propose this kind of methodology, which is both inclusive and neutral, may help inform public health strategies and individually tailored interventions.

The pandemic has led to significant changes in daily routines and lifestyle worldwide and, aside from resulting mental health issues, there have been reports of sleep disturbances in the general population during lockdowns. Circadian misalignment and sleep disruption have a profound impact on immune function and subsequently, the ability of individuals to combat infections. Xiaodong Zhuang, Zulian Liu and Sharlene Ting [18] summarize the evidence on the interplay between circadian biology, sleep and COVID-19 with the aims to identify areas of translational potentials—such as the optimum time to deliver drugs or vaccines, along with improving sleep quality—that may inform diagnostic and therapeutic strategies.

This issue of *Interface Focus* also ponders the lessons of previous pandemics for COVID-19. Infectious diseases have wreaked havoc on human communities since ancient times, shaping society as deeply and surely as revolution, war and economic crashes [19].

Past pandemics always pose questions about how we should act in future. Katie Dabin examines what the COVID-19 project is telling us about the impact of COVID-19 on cancer research and the collateral damage of the latest outbreak on future care [20]. She focuses on cancer treatment and research that will be featured in a new exhibition opening in October 2021, *Cancer Revolution: Science, Innovation and Hope,* at the Science and Industry Museum in Manchester. The exhibition has been updated despite COVID-19 restrictions to include stories and perspectives about the impact of the pandemic on individuals, health services and in research. The response to both underlines the necessity of global, collaborative research and the altruism of patients willing to participate in research—even when they might not experience the benefit of it themselves. Humans are, after all, the most cooperative species of all [21].

Natasha McEnroe and Stewart Emmens [22] describe the challenges faced by curators of the history of medicine when trying to collect and preserve objects that convey the impacts of COVID-19 while in the grip of the pandemic. As the gap between events and collecting has closed, with the rise of so-called ‘rapid response’ collecting, they also ask if it has become more subject to the whims and interests of the curator. Collecting in these challenging circumstances can also highlight existing issues, notably how to collect digital material, which ranges from apps and tweets to emails, pandemic modelling software and satirical social media.

Museum collections can provide important context. McEnroe and Emmens examine why the 1918–1919 influenza pandemic left behind so little material culture, in contrast with polio and tuberculosis. Perhaps this reflects how people did not want to be reminded of the trauma and death, perhaps much of the equipment continued to be used, or perhaps it was thought highly infectious, and discarded.

Britain a century ago mounted a relatively ineffectual response and Mark Honigsbaum argues there is a parallel in the way that in early 2020, as in 1918, medical professionals, and public health administrators, commentators and government advisors and politicians deprecated the severity of the coronavirus outbreak and, rather than screen travellers at the border and introduce community testing and rigorous contact tracing and quarantines, advised individuals with symptoms of coronavirus infection to self-isolate at home [23]: ‘Why this was the case will keep historians and committees of inquiry occupied for years’. He talks of a collective failure of imagination when it comes to envisaging how quickly our world could be thrown into turmoil by a new pandemic virus.

Though modelling is a crystal ball, often its vision can be cracked and clouded by incomplete data and understanding. Roy Anderson, Carolin Vegvari, Deirdre Hollingsworth, Li Pi, Rosie Maddren, Chi Wai Ng and Rebecca Baggaley examine how modelling has fared in the pandemic, where uncertainties remain in key areas such as the determinants of what predisposes to asymptomatic infection, what population fraction is asymptomatic, what is the infectiousness of such individuals, compared to those with symptoms, and how these are influenced by various variables [24].

They point out that, given the high transmissibility of the Delta variant which has spread rapidly worldwide, and in the light of data on breakthrough infections in a small proportion of vaccinated individuals, the concept of a target level of herd immunity by vaccination is no longer valid. To eradicate transmission, 100% of large populations would have to be effectively immunized to prevent continued transmission and the logistics required to achieve universal coverage are daunting, even in countries with robust healthcare infrastructure. More support from the richer nations is needed in resource poor settings since unvaccinated populations create opportunities for viral evolution.

Christl Donnelly and Ruth McCabe explore a key detail of the interface between science and society in the COVID-19 era by examining the development, communication and influence of mathematical transmission modelling to explore the public health impacts of the pandemic, and how to mitigate them with lockdowns and other interventions [25]. Unusually, as well as reviewing the models themselves, they draw on the opinions and experiences of modellers, scientific advisers, such as attendees of SAGE, SPI-M and comparable advisory bodies during the pandemic, and experts in science communication in the UK to explore and understand the complex relationships between models, decision-making, the media, and the public.

The study highlights the desire for increased two-way communication between these players, not least in conveying the extent to which the crystal ball of modelling is cracked. Complementing the points made by Whitty and Collet-Fenson, they point out how scientists must ensure that their models are of the highest scientific quality while also acknowledging their inherent limitations and the urgent need for results, while decision-makers must understand the nuances of underlying results and in the context of all other evidence.

The use of computer models to gaze into the future leads to the final theme of this issue of *Interface Focus*: how to visualize COVID-19, and the possible course of the pandemic by revealing the invisible worlds of the virus itself, along with how to envisage pandemic possibilities through the literature of science fiction.

Beginning with the former, Katy Barrett and Geoff Belknap [26] consider the history of image-making in medicine to trace how images have provided the means for discovery, for description and for diagnosis of disease and how these image-making practices are reflected in work to identify and visualize the Covid-19 virus in 2020–2021. They outline the different ways in which diseases have been located in the history of the medical image: in the community, in the body, in the cell, and on the image itself.

Starting from a contemporary art commission in the Science Museum's ‘Medicine: The Wellcome Galleries', they explore five examples of iconic medical images, by John Snow, Florence Nightingale, Arthur Schuster, and Donald Caspar and Aaron Klug, ending with a model of the coronavirus created by the MRC Laboratory of Molecular Biology in Cambridge. The latter underlines how one of the most striking visual results of the pandemic has been the coronavirus itself, whether in watercolours, or digitally, or in the form of a glass sculpture, the SARS-CoV-2 crown of spikes is now part of popular visual culture appearing in cartoons, artworks and even as an emoji. As they remark, no previous virus or disease has gained such visual currency as an image of the agent of pestilence and death.

Images of the virus have also benefitted from the maturation of a form of electron microscopy (EM). Kendra E. Leigh & Yorgo Modis [27] describe how the SARS-CoV-2 pandemic struck when recent advances in microscope hardware and analysis software, particularly in cryo-EM and cryo-electron tomography, have opened a new era in structural biology, making many previously intractable targets amenable to visualization to accelerate efforts to create COVID-19 vaccines and therapeutics.

One example is illustrated by how structures of the spike (S) glycoprotein of SARS-CoV-2 were available in March 2020, only a few months after the sequencing of the new virus. The spike structures underpinned the analysis of the effects of mutations, such as those shown by studies of other coronaviruses to improve immunogenicity, which could be used to hone SARS-CoV-2 vaccine formulations. Given the expectation of future pandemics given changes in land use, [28] they call for the momentum of this field to be sustained.

Finally, Glyn Morgan discusses how, in these unprecedented times, people turn to fiction as both a comforting distraction and a means to make sense of what lurks around the corner [29]. Fiction about pandemics and other disease outbreaks surged in 2020 and his survey of the long history of science fiction's engagement with disease demonstrates the ways in which these narratives, whether in literature or film, reflect contemporary cultural concerns. Ultimately, by portraying alternative worlds and possible futures, Morgan argues that science fiction can offer a ‘creative space’ to prompt new ways to think and learn. We need this space because it does not take a pandemic long to change the world: the time between the first international reports of a completely new infection, COVID-19, and the first UK wave was less than 12 weeks.

Museums can provide fresh perspectives on culture because they lie at the nexus of science, industry, the public and government, along with heritage, history, contemporary research and the future. In a similar way, this themed issue is a reminder that the most intriguing and thought-provoking insights often emerge at the interface between different disciplines, different eras, even different worlds.

## Data accessibility

This article has no additional data.

## References

[1] Morris PJT. 2010 Science for the nation: perspectives on the history of the Science Museum. London, UK: Palgrave Macmillan.

[2] Arnold K, Olsen D. 2020 Contagious cities. Sci. Mus. Gr. J. **14**, 10.15180; 201404. (10.15180/201405)

[3] Blatchford I. 2021 Vaccination and the Victorians: Lyon Playfair's battle for science. Science Museum Blog. https://blog.sciencemuseum.org.uk/vaccination-and-the-victorians-lyon-playfairs-battle-for-science/.

[4] Whitty CJM, Collet-Fenson LB. 2021 Formal and informal science advice in emergencies: COVID-19 in the UK. Interface Focus **11**, 20210059. (10.1098/rsfs.2021.0059) 34956605 PMC8504880

[5] Highfield R. 2020 Coronavirus: rise of mini-organs. Science Museum Group Blog. https://www.sciencemuseumgroup.org.uk/blog/coronavirus-rise-of-mini-organs/.

[6] Highfield R. 2020 Coronavirus: can AI solve future pandemics? Science Museum Group Blog. https://www.sciencemuseumgroup.org.uk/blog/coronavirus-can-ai-solve-future-pandemics/.

[7] Highfield R. 2021 Coronavirus: how to vaccinate a nation. Science Museum Group Blog. https://www.sciencemuseumgroup.org.uk/blog/coronavirus-how-to-vaccinate-a-nation/.

[8] Science Museum. 2021 Science Museum vaccination centre. https://www.sciencemuseum.org.uk/science-museum-vaccination-centre.

[9] Ball P. 2021 What the COVID-19 pandemic reveals about science, policy and society. Interface Focus **11**, 20210022. (10.1098/rsfs.2021.0022) 34956594 PMC8504882

[10] Phillips T, Zhang Y, Petherick A. 2021 A year of living distantly: global trends in the use of stay-at-home orders over the first 12 months of the COVID-19 pandemic. Interface Focus **11**, 20210041. (10.1098/rsfs.2021.0041) 34956599 PMC8504889

[11] Chinnery PF, Pearce JJ, Kinsey AM, Jenkinson JM, Wells G, Watt FM. 2021 How COVID-19 has changed medical research funding. Interface Focus **11**, 20210025. (10.1098/rsfs.2021.0025) 34956595 PMC8504879

[12] Smith JC, Goodhew DW. 2021 Personal observations on COVID-19 and the conduct and application of biomedical science. Interface Focus **11**, 20210053. (10.1098/rsfs.2021.0053) 34956604 PMC8504890

[13] Randall K, Ewing ET, Marr LC, Jimenez JL, Bourouiba L. 2021 How did we get here: what are droplets and aerosols and how far do they go? A historical perspective on the transmission of respiratory infectious diseases. Interface Focus **11**, 20210049. (10.1098/rsfs.2021.0049) 34956601 PMC8504878

[14] Greenhalgh T. 2021 Miasmas, mental models and preventive public health: some philosophical reflections on science in the COVID-19 pandemic. Interface Focus **11**, 20210017. (10.1098/rsfs.2021.0017) 34956591 PMC8504883

[15] Bhati AP et al. 2021 Pandemic drugs at pandemic speed: infrastructure for accelerating COVID-19 drug discovery with hybrid machine learning- and physics-based simulations on high performance computers. Interface Focus **11**, 20210018. (10.1098/rsfs.2021.0018) 34956592 PMC8504892

[16] Coveney PV, Dougherty ER, Highfield RR. 2016 Big data need big theory too. Phil. Trans. R. Soc. A **374**, 20160153. (10.1098/rsta.2016.0153) 27698035 PMC5052735

[17] Hampshire A, Hellyer PJ, Trender W, Chamberlain SR. 2021 Insights into the impact on daily life of the Covid-19 pandemic and effective coping strategies from free text analysis of people's collective experiences. Interface Focus **11**, 20210051. (10.1098/rsfs.2021.0051) 34956602 PMC8504891

[18] Liu Z, Ting S, Zhuang X. 2021 COVID-19, circadian rhythms and sleep: from virology to chronobiology. Interface Focus **11**, 20210043. (10.1098/rsfs.2021.0043) 34956600 PMC8504895

[19] Snowden FM. 2019 Epidemics and society. New Haven, CT: Yale University Press.

[20] Dabin K. 2021 Our mutual friends: cancer research in a time of COVID-19. Interface Focus **11**, 20210052. (10.1098/rsfs.2021.0052) 34956603 PMC8504881

[21] Nowak MA, Highfield R. 2011 Supercooperators: altruism, evolution, and why we need each other to succeed. New York, NY: Free Press.

[22] Emmens S, McEnroe N. 2021 Acquiring infection: the challenges of collecting epidemics and pandemics, past, present and future. Interface Focus **11**, 20210030. (10.1098/rsfs.2021.0030) 34956598 PMC8504894

[23] Honigsbaum M. 2021 Imagining pandemics now, and then: a century of medical failure. Interface Focus **11**, 20210029. (10.1098/rsfs.2021.0029) 34956597 PMC8504896

[24] Anderson RM, Vegvari C, Hollingsworth TD, Pi L, Maddren R, Ng CW, Baggaley RF. 2021 The SARS-CoV-2 pandemic: remaining uncertainties in our understanding of the epidemiology and transmission dynamics of the virus, and challenges to be overcome Interface Focus **11**, 20210008. (10.1098/rsfs.2021.0008) 34956588 PMC8504893

[25] McCabe R, Donnelly CA. 2021 Disease transmission and control modelling at the science–policy interface. Interface Focus **11**, 20210013. (10.1098/rsfs.2021.0013) 34956589 PMC8504885

[26] Barrett K, Belknap G. 2021 Locating disease spread: cholera to coronavirus and the art of the image. Interface Focus **11**, 20210014. (10.1098/rsfs.2021.0014) 34956590 PMC8504888

[27] Leigh KE, Modis Y. 2021 Imaging and visualizing SARS-CoV-2 in a new era for structural biology. Interface Focus **11**, 20210019. (10.1098/rsfs.2021.0019) 34956593 PMC8504884

[28] Gibb R et al*.* 2020 Zoonotic host diversity increases in human-dominated ecosystems. Nature **584**, 398-402. (10.1038/s41586-020-2562-8) 32759999

[29] Morgan G. 2021 New ways: the pandemics of science fiction. Interface Focus **11**, 20210027. (10.1098/rsfs.2021.0027) 34956596 PMC8504887

